# Relationships between key functional traits of the waterlily *Nuphar lutea* and wetland nutrient content

**DOI:** 10.7717/peerj.7861

**Published:** 2019-10-17

**Authors:** Charles P. Henriot, Quentin Cuenot, Lise-Hélène Levrey, Christophe Loup, Landry Chiarello, Hélène Masclaux, Gudrun Bornette

**Affiliations:** 1 Université de Franche-Comté, UMR CNRS 6249 Chrono-environnement, Besançon, France; 2 École Polytechnique Federale de Lausanne, Lausanne, Suisse; 3 Université Claude Bernard (Lyon I), Villeurbanne, France

**Keywords:** *Nuphar*, Macrophyte, Plant traits, Phenology, Wetlands, Eutrophication

## Abstract

Little attention has been paid to how aquatic habitat characteristics affect the traits of plant species. *Nuphar lutea* (L.) Sm. is a keystone species distributed across temperate regions of Europe, northwest Africa and western Asia. Its apparently low phenotypic variability compared to other aquatic plants led us to test whether the species exhibited significant phenotypic variability and whether trait values correlated to environmental parameters. The hypotheses were that (1) the environmental variation within our set of wetlands (both water and sediment characteristics) led to significant variation among four sets of traits related respectively to growth, reproduction, defence and storage and (2) that nutrient limitation (nitrogen and especially phosphorus) should affect plant traits towards a higher investment in storage and defence and a lower investment in growth and reproduction, thereby negatively affecting the success of *N. lutea*. To test these hypotheses, 11 populations of *N. lutea* were sampled in wetlands differing in physicochemical characteristics and spread along three rivers. A total of 15 traits, grouped into four sets (growth, reproduction, storage and defence), were measured during the growing season. Most *N. lutea* traits were related to the environmental characteristics of wetlands. The growth and reproduction traits were mostly positively related to habitat resource conditions, whereas the defence traits were positively correlated with both ammonium concentration and temperature, outlining possible anoxic stress (habitat adversity). Nitrogen or phosphorus limitation led to the variation of only a few traits: the rhizome starch content was higher in phosphorus-limited wetlands, while the rhizome length and volume, and the number of flowers were higher in nitrogen-limited wetlands.

## Introduction

Interactions between organisms and their environment, and the way environmental pressures affect plant traits, have been widely studied in terrestrial ecology in contrasting environmental situations (e.g. in various climatic and anthropic contexts, Jump & Penuelas, 2005) and at various spatial scales (e.g. from the plant scale to the biome, Gross et al., 2000). Among environmental constraints, resource limitation (fertility), disturbances (Lavorel & Garnier, 2002), and interactions with other plant or animal species (Sahli & Conner, 2011) have been investigated. It has been demonstrated that plant performance (e.g. size, growth rate) usually increases when habitat severity decreases (Chapin, Autumn & Pugnaire, 1993; McDonald, 1994; Puijalon & Bornette, 2004). At the species scale, plant traits usually vary in a consistent way when individuals face the same habitat features (Urbas & Zobel, 2000; Bret-Harte et al., 2001).

Aquatic habitats differ from terrestrial ones, as fluctuations of certain habitat conditions may vary more strongly in aquatic environments (e.g. water level fluctuations, attenuation and even stabilisation of temperatures, limitation of gas diffusion, carbon and gravity limitation, Bornette & Puijalon, 2011; Rascio, 2002). As a consequence, aquatic plants are considered to have a very high ability to adjust their traits to habitat variation, leading either to high phenotypic plasticity or to local adaptation (Wells & Pigliucci, 2000; Santamaría, 2002). The way population traits vary along environmental gradients has been considered (Titus & Sullivan, 2001; Dorken & Barrett, 2004), but the variation of a large set of plant traits has rarely been included. Some studies focused on trait variation along environmental gradients, such as alkalinity (Kok, Van Der Velde & Landsbergen, 1990), sediment quality or water depth (Titus & Sullivan, 2001), current velocity (Puijalon, Bornette & Sagnes, 2005) or nutrient level (Keddy, Gaudet & Fraser, 2000). However, such studies rarely simultaneously assess many traits related to major plant functions and thus fail to identify whether habitat adversity decreases the global population performance or leads to compromises between functions. This information is particularly essential for the development of tools to preserve aquatic plant biodiversity that is under great threat from global change through eutrophication, increase in temperatures, decrease in aquatic connectivity between habitats, and decrease in habitat areas, diversity and abundance.

Among aquatic plants, species of Nymphaeaceae are singular because of their basal and partly non-elucidated position in angiosperm phylogeny (Padgett, Les & Crow, 1999; Du & Wang, 2016) and their very ancient occurrence in the fossil register (Du & Wang, 2016). Their rather unique and very large size among strictly aquatic plants and their dominance in quite monospecific stands in undisturbed ecosystems (Bornette et al., 2008) make them a major contributor to aquatic plant biomass (considering both above and belowground biomass) and turn-over (Brock, Van Der Velde & Van de Steeg, 1987). These species are able to impede phytoplankton growth, both directly (through their shading effect) and indirectly (through their ability to act as refuges for zooplankton that grazes phytoplankton). They are consequently able to hamper phytoplankton growth in eutrophic water bodies (Kornijów, Measey & Moss, 2016). Furthermore, such floating-leaved aquatic plants are able to tolerate oxygen shortage stress by a ventilation system that operates through rhizomes and leaves (Dacey, 1981; Dacey & Klug, 1982). The convective through-flow in these aquatic plants is the result of a gas-pumping system, commonly powered by solar radiation, which improves the internal aeration of submerged plant organs (Grosse, Büchel & Tiebel, 1991; Große, 1996; Ribaudo et al., 2012), and confers beneficial effects on the ecosystem (e.g. increased oxygen transfers to rhizosphere, root growth and nutrient uptake, Strand & Weisner, 2002; Nakamura et al., 2013). *Nuphar lutea* is a representative of this group. This keystone species is distributed in the temperate regions of Europe, northwest Africa and western Asia (Padgett, 2007). *N. lutea* possesses floating and submerged leaves and an extensive rhizome bearing the roots which can penetrate deep in the sediment. Its peduncles produce only one flower at the top and fruits above or floating on the water surface. *N. lutea* colonises very contrasting habitats in terms of trophic level, current velocity, alkalinity, etc. (Van Der Velde, Custers & De Lyon, 1986; Smits et al., 1988; Padgett, Les & Crow, 1999). Its very large size compared to other aquatic taxa and its very long life span due to its clonal growth, its thick and large rhizomes, and its late maturity (Barrat-Segretain, 1996) place it as a top-competitor among aquatic plants (Bornette et al., 2008). Its high drag reduces local flow velocities and promotes sedimentation, including organic matter deposition, creating a high-nutrient and low hydrodynamic environment (Puijalon et al., 2011; Schoelynck et al., 2014). These various functions make *N. lutea* a keystone species in freshwater ecosystems.

Even if the morphological variation of *N. lutea* is described in a few papers (Kouki, 1993; Puijalon et al., 2011; Schoelynck et al., 2014; Klok & Van Der Velde, 2017), *N. lutea* is generally considered to have poor morphological variation compared to other aquatic plants (Wells & Pigliucci, 2000). Thus, the way environmental conditions relate to major functions of *N. lutea* would provide important information about the persistence of this species through space and time.

The aim of this study was consequently to determine how four sets of traits of *N. lutea—*respectively related to (1) growth, (2) reproduction, (3) storage and (4) defence—and considered as components of fitness (Ackerly et al., 2000; Weiner, 2004) vary among wetlands differing in environmental conditions (temperature, water and substrate resources, alkalinity, phytoplankton abundance). Aquatic plant species exhibit optimal rates of photosynthesis at relatively high temperatures, and enhanced growth rates result from increased temperature (e.g. between 20 and 35 °C, Santamaría & Van Vierssen, 1997; or between 28 and 32 °C, Barko, Adams & Clesceri, 1986). Water temperature, therefore, may encourage the development and the expansion of macrophytes such as *N. lutea*. In addition to temperature, the concentration of ions in the water affects water pH, and consequently may affect the bioavailability of nutrients (carbon and phosphorus, for example, Maberly & Madsen, 2002; Smolders et al., 2006). The water ion concentration also rules the rates of photosynthesis of submerged macrophytes, and therefore their different traits (Maberly & Spence, 1989). Among nutrients, phosphorus and nitrogen are the more limiting compounds in aquatic ecosystems (Suttle & Harrison, 1988; Güsewell, Koerselman & Verhoeven, 2003). Nutrient availability increases competition and gives an advantage to tall competitive species and floating species (Bornette & Puijalon, 2011; Arthaud et al., 2012). Ultimately, nutrients (as nitrogen or phosphorus) may lead to a shift from macrophyte-dominated water stages to phytoplankton turbid water stages when fishes occur in the water-body (Jeppesen et al., 2000; Arthaud et al., 2013). Nitrogen, phosphorus and organic carbon concentrations in the sediment have been documented as potentially ruling macrophyte development (Bolpagni & Pino, 2017). Nutrients should thus favour the expansion of plants, that is, increase in growth and reproduction traits (Reddy, Agami & Tucker, 1990), whereas low nutrient availability could lead to a decrease their abundance, and increase the plant investment in storage (Reddy, Agami & Tucker, 1990; Puijalon, Piola & Bornette, 2008) and defence (Coley, Bryant & Chapin, 1985; Grime, 2006).

Therefore, we first explored whether environmental variation within our set of wetlands (both water and sediment characteristics) led to significant variation among these fitness-related traits (related to growth, reproduction, storage and defence). Concerning nutrient limitation, we expected that limitations in nitrogen and especially phosphorus (Suttle & Harrison, 1988; Güsewell, Koerselman & Verhoeven, 2003) should affect plant traits towards a higher investment in storage and defence and a lower investment in growth and reproduction (Kubin, Melzer & Čížková, 1994; Grime, 2006), and negatively affect the success of *N. lutea*.

## Materials and Methods

### Studied wetlands

**Figure 1:**
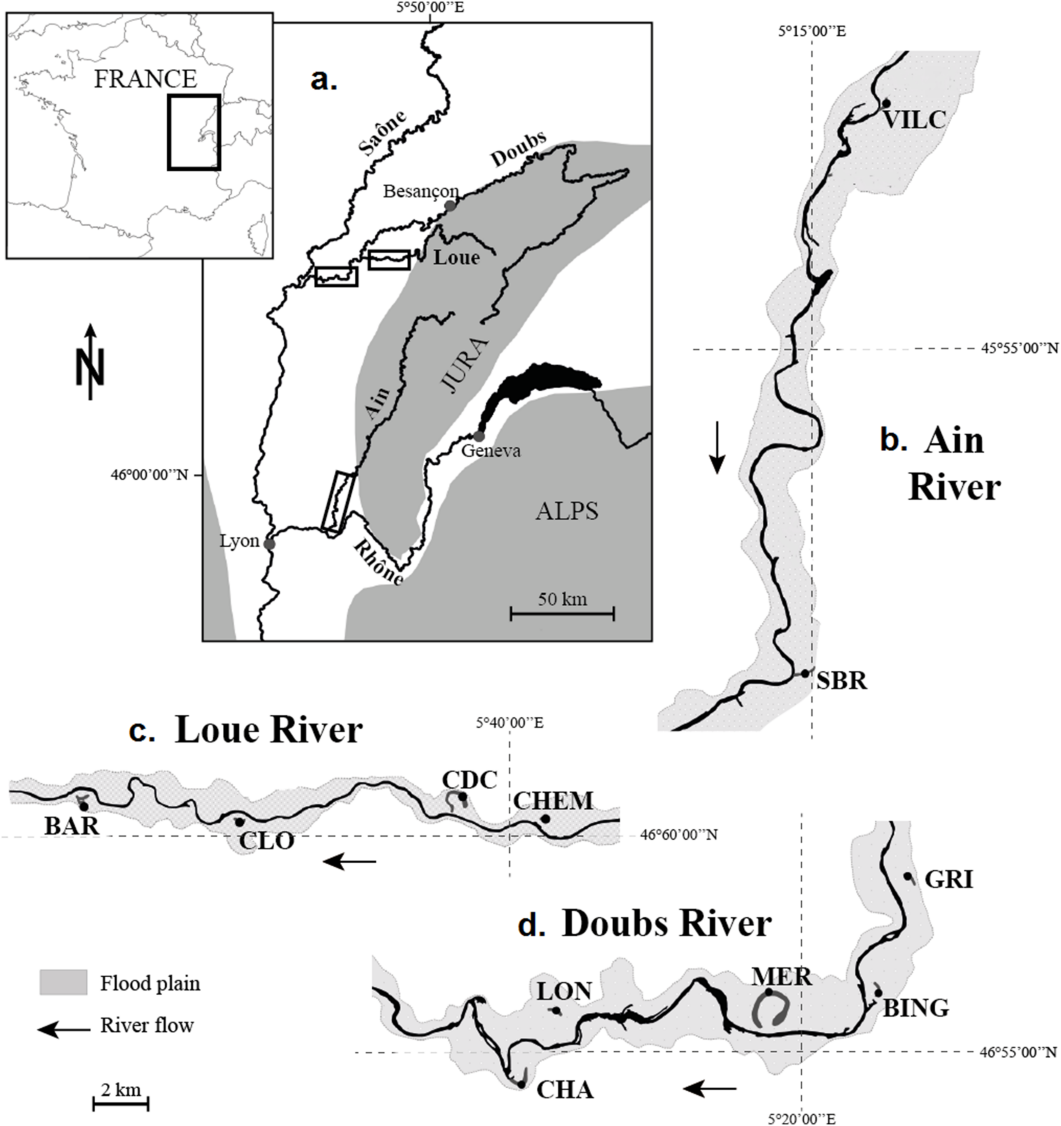
Location of the 11 studied wetlands. (A) Location of the river network under study on the map of France. (B) The Ain River. (C) The Loue River. (D) The Doubs River. (A) Location of the river network under study on the map of France; boxes indicate the location of the river reaches shown in (B–D). (B) Ain River, from upstream to downstream: VILC (Vilette Centre) and SBR (Sous-Bresse). (C) Loue River, from upstream to downstream: CHEM (Chemillière), CDC (Chemain des Creux), CLO (Le Clos) and BAR (Le Baraquier). (D) Doubs River, from upstream to downstream: GRI (Grimonts), BING (Bras des Inglas), MER (Meraton), LON (Longepierre) and CHA (Charette).

The study focused on 11 populations of *N. lutea* collected in 11 wetlands dispatched along three rivers in the eastern part of France: the Ain, the Doubs and the Loue Rivers, all three of which belong to the Rhône watershed and flow on the calcareous Jura massif (Fig. 1). The wetlands were selected to maximise habitat contrast (mainly for nutrient loads and temperature) and because populations of *N. lutea* were sufficiently abundant, so that the sampling effort had a negligible impact on the population size at the wetland scale. In Doubs and Loue wetlands, *N. lutea* stands were rather monospecific, whereas in the Ain wetlands, *N. lutea* stands were surrounded by other aquatic plant patches (*Callitriche platycarpa, Berula erecta, Mentha aquatica*, *Sparganium emersum*, *Potamogeton natans*, *Groenlandia densa*, *Myriophillum verticillatum*).

### Physicochemical characteristics of water

To assess whether the habitat conditions in wetlands affect plant traits, we documented monthly over a year cycle, from April 2015 to March 2016, the water parameters that may directly (nutrients, temperature) and indirectly (dissolved oxygen, phytoplankton abundance, dissolved organic carbon (DOC)) influence *N. lutea* traits. The physicochemical dataset, therefore, resulted in nine physicochemical parameters, measured once a month for 12 months, in the 11 studied wetlands.

The water temperature was measured automatically using submerged probes (HOBO UTBI-001 Tidbit; Onset Computer Corporation, Bourne, MA, USA) that registered the water temperature each day at 12:00 AM (UTC). The probes were fixed at a few metres from the 11 *N. lutea* populations, leaving them accessible for monthly surveys without disrupting the development of the studied populations (i.e. just outside the stand of *N. lutea* populations).

The other physicochemical parameters were recorded each month in each wetland near the temperature probes. We measured the electrical conductivity in situ at a 10-cm depth and the pH using a conductivity WTW sensor (TetraCon^®^ 325; Onset Computer Corporation, Bourne, MA, USA) and a pH WTW sensor (SenTix^®^ 41; Onset Computer Corporation, Bourne, MA, USA), respectively. Dissolved oxygen in water is ruled by photosynthetic activity, water motion and oxygen demand for organic matter mineralisation. Consequently, we documented monthly oxygen (WTW sensor, CellOx^®^ 325-3; Onset Computer Corporation, Bourne, MA, USA), DOC and chlorophyll-a concentrations in water.

Concentrations in nitrate and ammonium nitrogen, phosphate and DOC of the water were measured in the laboratory. For this purpose, at each sampling date, 500 ml of water was collected, brought back to the laboratory in a cooler, filtered the day of collection (polycarbonate filters with a mesh size of 0.2 µm), stored at 4 °C, and analysed within 48 h. The P-phosphate [P-PO_4_^3−^] concentration was measured using the molybdenum blue colouration method after acidification (Rodier et al., 1996). The N-ammonium [N-NH4^+^] concentration was measured with the indophenol blue method (Berthelot’s reaction, Merck Spectroquant 14752). The N-nitrate [N-NO3^−^] concentration was measured using a Dionex ICS-1000 ion chromatograph. The DOC concentration was measured with a TOC analyser (Vario TOC Cube; Elementar, Langenselbold, Germany). To assess the chlorophyll-a concentrations, three liters of water were sampled and filtered through a 200-µm filter to remove zooplankton and any detritus and further filtered through 0.7-µm mesh filters, which were stored at −20 °C until extraction (overnight at 4 °C in acetone (90%)). Chlorophyll-a concentrations were measured the next day using a spectrophotometer (calculation taking into account measures done with λ = 630, 645 and 663 nm; Yentsch & Menzel, 1963). Dissolved oxygen in water was measured in situ 10 cm below the water surface for each sampling date using a WTW oxygen sensor.

The averaged values obtained for each parameter are grouped in Table S1.

### Nutrient loads of wetland sediment

We measured nitrogen, phosphorus and organic carbon sediment concentrations in the 11 wetlands. For this purpose, we sampled sediment using a nine cm-diameter sediment corer just outside the stands of *N. lutea* in November and December 2014. The nitrogen and phosphorus concentrations were analysed (i) in the upper fraction of two centimeters of the sediment core because of the high bioavailability of nutrients and the high nutrient exchanges between water and sediment in this layer, and (ii) in the 0–40 cm fraction of the sediment core to take into account all the nutrients accessible by the roots of *N. lutea*. Each sediment sample was measured for total phosphorus (inductively coupled plasma—atomic emission spectrometry, iCAP6500 Radial; Thermo Fisher, Waltham, MA, USA), nitrogen (thermic catalytic oxidation, thermic conductivity detection, macro analyser Variomex; Elementar), and organic carbon concentration (thermic catalytic oxidation, infra-red detection, Vario TOC Cube; Elementar, Langenselbold, Germany).

The averaged values obtained for each nutrient are grouped in Table S2.

### Plant traits sampling and analyses

Traits related to four sets of traits (growth, reproduction, defence and storage) were sampled (Table 1). Concerning growth, plant size is frequently used as a reference trait for terrestrial plants. Despite their strong indicative value, traits related to plant size are rather difficult to quantify for aquatic plants, and more specifically, for *N. lutea*. Indeed, plant height is controlled by water depth. Moreover, the individual size is frequently impossible to assess because of the very large size of *N. lutea* clones due to its extensive rhizomes, which are usually several metres long, branched in all directions, intermingled and strongly and deeply anchored in the underwater sediment. Furthermore, we wanted to relate plant traits to the environmental strains recently encountered by the plant. For this reason, we selected plant traits independent from water depth and individual long-term history and focused on traits related to recent growth of the plant, informing on the conditions it encountered during or just before habitat sampling. The rhizome length produced per apex per year and the number of leaves produced per apex per year were consequently selected to qualify the growth of *N. lutea* and documented on the part of the rhizomes corresponding to the last growing year. For reproduction, we documented the number of flowers produced per apex per year (counting the flower peduncle scars in rhizomes, as described later in the ‘Rhizome traits’ section), the number of seeds produced per fruit, the fruit water content and the seed:fruit mass ratio. For defence, we focused on floating leaf blades (which face high grazing pressure, Stenberg & Stenberg, 2012; Klok & Van Der Velde, 2019), because submerged leaves were absent of many sites, particularly the eutrophic ones (despite the fact that submersed leaves may be more consumed by herbivores as ducks and swans, Cronin, Wissing & Lodge, 1998). For this purpose, the leaf water content (i.e. the floating leaf blades water content) and the C:N ratio, related to the tissue cost and induced resistance (nitrogen enrichment favour the production of low-density tissues, and reduced nitrogen availability induces the production of high-density tissues rich in lignin and structural carbohydrates, Garnier & Laurent, 1994; Poorter & De Jong, 1999; Craine et al., 2001) were measured. Sclereid abundance was also measured because it is considered, as high density tissues, to prevent grazing and increase plant rigidity (Konno, Inoue & Nakamura, 2014). For storage, macrophytes accumulate starch in their rhizomes (Kubin, Melzer & Čížková, 1994). We measured the rhizome starch and water contents. Starch accumulation in rhizomes potentially leads to the increase of rhizome diameter, and so, the diameter and the volume of the rhizomes were also measured.

**Table 1:** Correspondence between the plant traits and the four sets of traits studied, and sampling pressure (number of samples collected in a given wetland, sampled in different individuals) for each trait. Only three and five fruits were sampled for VILC and SBR respectively, because of the low fruit availability likely due to strong herbivory pressure.

Organ sampled	Sampling pressure	Trait	Unit	Corresponding set of traits	Trait related information
Rhizomes	5	Rhizome volume	cm^3^	Storage	Size of the storage organ
		Rhizome water content	% FM	Storage	Density of the storage organ
		Rhizome diameter	mm	Storage	Size of the storage organ
		Rhizome length	mm	Growth	Growth rate
		Starch content	% DM	Storage	Quantity of stored reserves
		Leaf number	–	Growth	Growth rate
		Flower number	–	Reproduction	Investment in reproduction
Leaf blades	5	C:N ratio	–	Defence	Tissue cost and defence
		Sclereid abundance	number per cm²	Defence	Increase plant rigidity and decrease grazing pressure
		Leaf water content	% FM	Defence	Tissue cost and defence
Fruits	10 (except VILC and SBR)	Seed number per fruit	–	Reproduction	Reproductive success
		Fruit water content	% FM	Reproduction	Combined with seed/fruit ratio, quality of seeds
		Seed:fruit mass ratio	–	Reproduction	Reproductive success

**Note:**

FM, Fresh Mass; DM, Dry Mass.

Plant samples were collected in the field on June 23, 2015 for the Doubs and the Loue Rivers and on June 24, 2015 for the Ain River. For each wetland, we cut the end of five growing rhizomes (approximately 80 cm length, including apex and the following rhizome), five fully developed undamaged floating leaf blades and 10 full-grown fruits were randomly sampled in the population (Table 1). The number of plants sampled per wetland was a compromise between (1) the duration of the data sampling for a given population (trait documentation being time-consuming and having to be documented rapidly after the sampling), (2) the necessity to collect rhizomes being at the same growth stage in the sampling season and (3) the fact that sampled wetlands were located far from each other. The plants were collected on a circle whose centre was the water sampling point. Each plant sample was collected at a minimum distance of 10 m from each other. When the population was discontinuous, a sample was collected in each plant patch (VILC and VILM wetlands). Rhizome fragments were collected in order to obtain the apex and at least 3 years of growth. The first fully developed and mature floating leaf blades from the centre of the apex were sampled (which may reduce strongly the developmental effect on sclereid abundance; see below). For the wetlands of the Ain River, only three and five fruits were sampled for VILC and SBR, respectively, because of the strong herbivory pressure on flowers and fruits (many peduncles were found without fruits, possibly consumed by ducks or swans). The *N. lutea* traits dataset, therefore, results in 13 traits, randomly measured once in five or 10 replicates (depending on the trait; see above), in the 11 studied wetlands.

#### Rhizome traits

The rhizomes of *N. lutea* provide information about the growing history of the plant because the leaves and flowers leave scars when they die. These scars are ranged along several spirals (usually three) all along the rhizome. During summer blooming, *N. lutea* flowers by producing a maximum of one flower per spiral per year. Thus, the occurrence of flower peduncle scars indicates the summer period along the rhizome growth, and peduncle scars allowed us to divide the rhizome into sections corresponding to years. As the rhizomes were collected in June 2015, that is, during the growing season, we could not quantify the 2014–2015 growth. We thus measured the rhizome growth by measuring the rhizome length produced during the 2013–2014 year, that is, the rhizome length between the last 2013 peduncle scar and the last 2014 peduncle scar. Similarly, for each rhizome, we counted flower peduncle and leaf petiole scars produced after the last 2013 peduncle scar and before the last 2014 peduncle scar. The volumes of rhizomes were essentially calculated as volumes of cylinders, using the lengths and the diameters corresponding to the 2014 and 2013 years. We compared the peduncle and petiole scars, and the rhizome dimensions for 2014 and 2013 using a simple Student *t*-test. All *p*-values showed that theses metrics did not differ significantly between two consecutive years. Moreover, the flows of the three rivers did not differ between 2014 and 2015 (*t*-test, data from the Ministry of Ecology, Sustainable development and Energy). We considered consequently that the growth parameters measured, corresponding to 2014, were representative of the physicochemical conditions of the wetlands measured in this study (from April 2015 to March 2016). The starch content of rhizomes was measured on dry rhizome sections corresponding to the 2014 year using the Megazyme test kit procedure (Megazyme International Ireland Limited, Bray, Ireland). As the starch content was measured in the recently produced rhizome, even if it was documented in spring, the stored reserve may be modulated by accumulation, mobilisation, and redistribution of starch, and the starch content of the rhizome can be underestimated (Kausch, Seago & Marsh, 1981; Grasset et al., 2015). The water content of the rhizomes was obtained as follows: first, the fresh mass was weighed. Then, the rhizomes were dried at 100 °C for 24 h, and the dry mass (DM) was measured. Finally, the water content was obtained using the following equation:

(1) $$H\left( \% \right) = {{{\rm{FM}} - {\rm{DM}}} \over {{\rm{FM}}}} \times 100$$

#### Leaf traits

Floating leaf blades without petioles were frozen at −80°C and lyophilised for 24 h. The leaf water content was obtained using Eq. (1). Dried floating leaf blades were crushed in liquid nitrogen. The shredded floating leaf blades were passed through a 0.5-mm sieve to remove the coarse elements. It was then passed in a carbon-nitrogen elementary analyser to obtain the C:N ratio of the floating leaf blades. To quantify the sclereid abundance, histological sections of the petiole just below its insertion in leaf blade were coloured following the Tolivia and Tolivia protocol (Tolivia & Tolivia, 1987) with Safranin O and Alcian Blue. In sections, sclereids were observed by the aid of the binocular loupe and expressed per cm^2^. To have a common area of reference for all the microscope slides (i.e. for each floating leaf blade), only the sclereids visible on the cutting plane were counted. Three petiole sections were made per floating leaf blade, and the results obtained for the three replicates were averaged to obtain an average number of sclereids per cm^2^.

#### Fruit traits

For each fruit collected, seeds were extracted and counted using the WinSeedle software (Regent Instruments Inc., Sainte-Foy, QC, Canada). Fresh seed and fruit tissues were weighed and then dried separately in an oven at 60 °C for 12 h to obtain their dry masses and the fruit water content (1). The average seed DM was obtained by dividing the whole seed mass of the fruit by the number of seeds. The seed:fruit mass ratio was calculated as the ratio of the average DM of a seed and of the DM of the corresponding fruit.

The averaged values obtained for each trait are grouped in Table S3.

### Statistical analyses

The normality and homoscedasticity of the data were tested using the Shapiro–Wilk, Levene and Bartlett tests. When necessary (rhizome volume), the data were log-transformed to reach normality. The number of flowers per year, which followed a Poisson model, was not included in linear models (LM) (see below).

A principal component analysis (PCA) was first performed to discriminate wetlands according to the physicochemical characteristics of the water. The median values of the 12 measures of each of the water physicochemical characteristics (1/month) were considered in the PCA. The scores of each water physicochemical characteristic on the PCA axes were verified using bootstrapping. Bootstraps were replicated 10,000 times, and bootstrap confidence intervals of 95% were constructed. Then, to identify the potential role of the wetland physicochemical characteristics on *N. lutea* traits, we performed LM. LM related traits (except the number of flowers) with (i) the coordinates of the 11 wetlands along the two first axes of the PCA of the physicochemical characteristics of water, and with (ii) the nitrogen and phosphorus sediment concentrations (the organic carbon was removed from this analysis because it was highly correlated with the sediment nitrogen, *R*^2^ = 0.87 for the upper two centimeters and *R*^2^ = 0.77 for the upper 40 cm). Because they are counts, the number of flowers, leaves and seeds were analysed with generalised linear models (GLM). Because the variance of the seed number is much larger than its mean, a negative binomial GLM was used for this trait. The number of leaves and seeds was therefore analysed with both LM and GLM.

Some authors proposed that the ecosystems may change from nitrogen-limited functioning to phosphorus-limited functioning depending on the value of the N:P ratio in plant tissues (N/P < 18 and N/P > 24, Suttle & Harrison, 1988; N/P = 20, Güsewell, Koerselman & Verhoeven, 2003). We tested whether this trophic partitioning of ecosystems may lead to significant differences in plant traits. These thresholds led to the same wetland partitioning: four wetlands (GRI, CHA, LON, CHEM) had N:P ratios <20 and were considered as nitrogen-limited (i.e. led by the phosphorus), while the seven others had N:P ratios >30 and were considered as phosphorus-limited (i.e. led by the nitrogen). Some boxplots were performed to determine the potential consequences of the ecosystem trophic functioning on traits of *N. lutea*. The significance of the difference between the two groups was tested thanks to a *t*-test for each trait.

The ‘Holm sequentially rejective Bonferroni test’ (Holm, 1979) was used to adjust the *p*-values of all tests and to counteract the problem of multiple comparisons. It was used because it is considered to be more powerful than the Bonferroni correction (Holm, 1979; Abdi, 2010). However, because the Holm adjustment inflates the type 1 error (Abdi, 2010), we decided to keep both the original and corrected *p*-values.

The α-value was set to 0.05.

All the analyses were done using RStudio (Version 1.0.153) and the R package (R Core Team, 2017).

## Results

### Physicochemical characteristics of wetlands

**Figure 2:**
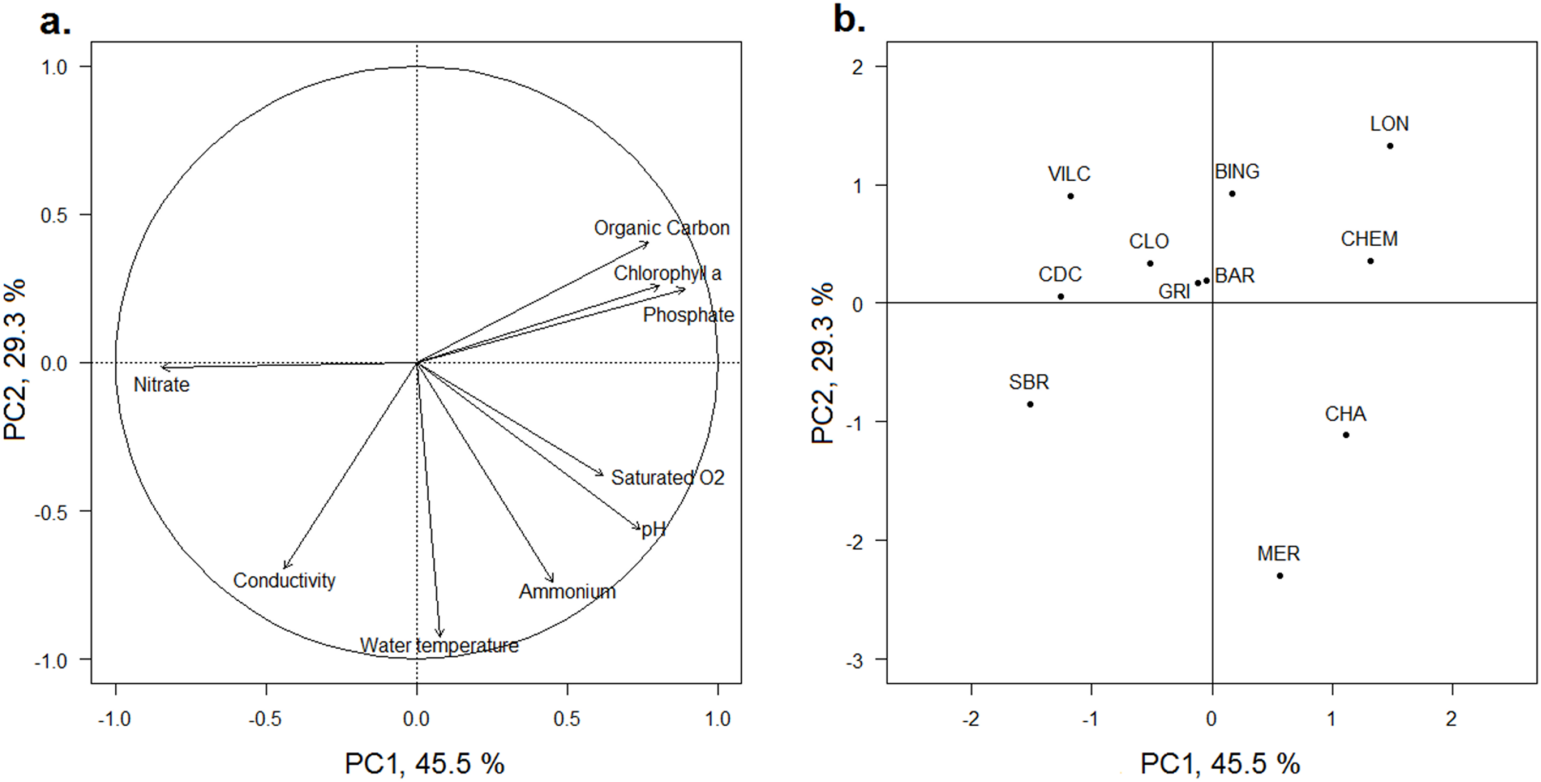
PCA of the physicochemical characteristics of the water of the 11 studied wetlands. (A) Correlation circle of the factorial coordinates of the physicochemical parameters with the two first axes of the PCA. (B) Factor map of the factorial coordinates of wetlands along the two first axes of the PCA. The wetland codes and the wetland locations are indicated in Fig. 1.

The first and second axes of the PCA (Fig. 2) explained 45.5% and 29.3% of the variance of the physicochemical dataset, respectively (Table S1). All scores of each water physicochemical characteristic on the PCA axes were within the bootstrap confidence intervals of 95%. The first axis (PC1) was mainly positively correlated with phosphates, chlorophyll-a and organic carbon concentrations in water and negatively with the nitrate concentration and to a lesser extent, to conductivity (Fig. 2A). Both Ain and Loue wetlands (CLO, VILC, CDC and SBR) were plotted on the negative part of the axis, whereas three Doubs wetlands (CHA, CHEM and LON) were plotted on the positive part of the axis (Fig. 2B). The PCA separated wetlands according to the nutrient concentration in water (PC1), and negative values were related to wetlands with higher nitrate concentrations (0.008 mg.l^−1^< [N-NH_4_] < 0.027 mg.l^−1^; 1.11 mg.l^−1^< [N-NO_3_] < 4.99 mg.l^−1^; 0.006 mg.l^−1^< [P-PO_4_] <0.014 mg.l^−1^ for CLO, VILC, CDC and SBR), while positive values were correlated with wetlands having higher phosphate and ammonium concentrations (0.020 mg.l^−1^ < [N-NH_4_] < 0.039 mg.l^−1^; 0.80 mg.l^−1^ < [N-NO_3_] < 1.17 mg.l^−1^; 0.046 mg.l^−1^ < [P-PO_4_] < 0.087 mg.l^−1^ for CHA, CHEM and LON) (Table S1). The second axis (PC2) was negatively correlated with temperature, ammonium concentration and conductivity and to a lesser extent, with the pH and dissolved oxygen (Fig. 2A). Most wetlands were plotted on the positive part of this axis, but two large oxbow lakes (CHA and MER, which had higher average temperatures of 15.1 °C and 14.5 °C, respectively) and ammonium nitrogen concentrations in water (0.038 and 0.039 mg.l^−1^, respectively) were plotted on the negative side of the axis (Fig. 2B). No general grouping by river was visible.

Concerning the sediment nutrient concentrations, three wetlands (MER, CDC and CLO) had high nitrogen and organic carbon concentrations (positively correlated between each other, *R*^2^ = 0.87 for the upper two centimeters and *R*^2^ = 0.77 for the upper 40 cm). The phosphorus sediment concentration was higher in CHA compared to the other wetlands. These results were the same for the two considered sediment layers (both 0–2 and 0–40 cm layers).

### Relationships between plant traits and physicochemistry of wetlands

LM and GLM results were congruent between each other considering traits analysed by the two modelling methods (leaf and seed numbers, Table 2).

**Table 2:** Results of the LM and GLM performed between the plant traits and (i) the two first axes (respectively PC1 and PC2 axes) of the PCA of the physico-chemical characteristics of water, and (ii) the N and P concentrations of wetland sediments.

Trait	Trait group	Plant traits and water physico-chemical characteristics		Plant traits and sediment physico-chemical characteristics			
		*p*-values of the model—PC1 axis	*p*-values of the model—PC2 axis	*p*-values of the model—N concentration in the two upper cm of sediment	*p*-values of the model—P concentration in the two upper cm of sediment	*p*-values of the model—N concentration in the 40 upper cm of sediment	*p*-values of the model—P concentration in the 40 upper cm of sediment
**LM**							
Rhizome length	Growth	**0.010 (+)**	**0.042 (+)**	**0.0423 (−)**	**0.028 (+)**	0.257 (−)	**0.013 (+)**
Leaf number	Growth	0.486 (+)	0.115 (+)	0.508 (−)	0.248 (+)	0.784 (−)	0.189 (+)
Seed number	Reproduction	**1.1.10**^**−6**^ (+)*	0.159 (+)	**0.012 (−)**	0.068 (+)	**0.026 (−)**	**0.00395 (+)**
Fruit water content	Reproduction	0.214 (−)	0.921 (+)	0.730 (+)	0.647 (−)	0.217 (+)	0.433 (+)
Seed:fruit mass ratio	Reproduction	0.00317 (+)*	0.00265 (−)*	**0.039 (+)**	**2.12.10**^**−4**^ (+)*	0.150 (+)	**0.033 (+)**
Rhizome starch content	Storage	0.393 (+)	0.195 (−)	0.627 (+)	0.423 (+)	0.767 (+)	0.298 (+)
Rhizome water content	Storage	0.850 (−)	**0.049 (−)**	0.874 (−)	0.309 (−)	0.819 (−)	0.374 (−)
Rhizome diameter	Storage	**4.85.10**^**−4**^ (+)*	0.372 (+)	**0.021 (−)**	0.362 (+)	**0.017 (−)**	0.146 (+)
Rhizome volume	Storage	**2.21.10**^**−4**^ (+)*	0.111 (+)	**0.036 (−)**	0.101 (+)	0.073 (−)	**0.031 (+)**
Leaf C:N ratio	Defense	**1.70.10**^**−5**^ (+)*	**1.08.10**^**−5**^ (−)*	0.701 (+)	**0.00731 (+)**	0.373 (+)	**0.0094 (+)**
Leaf sclereid abundance	Defense	0.106 (+)	**0.010 (−)**	0.113 (+)	0.146 (+)	0.191 (+)	0.401 (+)
Leaf water content	Defense	0.156 (−)	0.00391 (+)*	0.487 (−)	0.222 (−)	0.591 (−)	0.398 (−)
**GLM**							
Leaf number	Growth	0.650 (+)	0.485 (+)	0.683 (−)	0.462 (+)	0.866 (−)	0.402 (+)
Flower number	Reproduction	0.00106 (+)*	0.195 (+)	**0.026 (−)**	0.435 (+)	**0.0166 (−)**	0.275 (+)
Seed number	Reproduction	**4.01.10**^**−6**^ (+)*	0.833 (+)	**0.026 (−)**	0.217 (+)	**0.048 (−)**	**0.022 (+)**

**Note:**

The signs (−) and (+) relate respectively to a negative or a positive relationship between the trait and the axis considered. Bolded entries are significant before the Holm correction of the *p*-values.

* Indicate relationships that remain significant after the Holm correction of the *p*-values.

Concerning the physicochemical characteristics of water, only the rhizome length was positively correlated to wetland coordinates along PC1 and PC2 among the growth traits (Table 2). Among reproduction traits, the number of flowers and seeds and the seed:fruit mass ratio were positively correlated to the wetland coordinates along PC1. The seed:fruit mass ratio was also negatively correlated to PC2 (Table 2). Among storage traits, the diameter and the volume of rhizomes were positively correlated to wetland coordinates along PC1 (Table 2). The rhizome water content was negatively correlated with the wetland coordinates along PC2. For defence traits, the sclereid relative abundance was negatively correlated and the leaf water content was positively correlated to the wetland coordinates along the second axis of the PCA, and the C:N ratio was correlated to both PC1 and PC2 (positive correlation on PC1 and negative correlation on PC2; Table 2).

After the adjustment of the *p*-values (Holm), all the traits but the rhizome length (on both PC1 and PC2), the rhizome water content (on PC2) and the sclereid relative abundance (on PC2) remained significantly related to water characteristics.

Concerning the sediment nutrient concentrations, the results were almost identical depending on the layer considered (both 0–2 and 0–40 cm layers; Table 2). The rhizome length was positively correlated with the phosphorus sediment concentration in both sediment layers, and negatively correlated with the nitrogen concentration in the upper two centimeters of sediment (Table 2). For the reproduction traits, the seed:fruit mass ratio was positively correlated with both the nitrogen and organic carbon sediment concentrations (upper two centimeters of sediment), and with the phosphorus sediment concentration (both sediment layers). The numbers of flowers and seeds were negatively correlated with both the nitrogen and organic carbon concentrations in both sediment layers (Table 2). The numbers of seeds was also positively correlated with the phosphorus concentration when considering the 0–40 cm sediment layers (Table 2). The rhizome diameter was negatively correlated with both the nitrogen and organic carbon sediment concentrations in both sediment layers (Table 2). The rhizome volume was negatively correlated with both the nitrogen and organic carbon in the upper two centimeters of sediment, and positively correlated with the phosphorus in the upper 40 cm of sediment (Table 2). Concerning the defence traits, only C/N was positively correlated with the phosphorus concentration in both sediment layers (Table 2).

After the Holm adjustment of the *p*-values, only the seed:fruit mass ratio remained correlated with phosphorus in the upper two centimeters of wetland sediments.

### Trait contrast according to the nitrogen or phosphorus trophic limitation of wetlands

**Figure 3:**
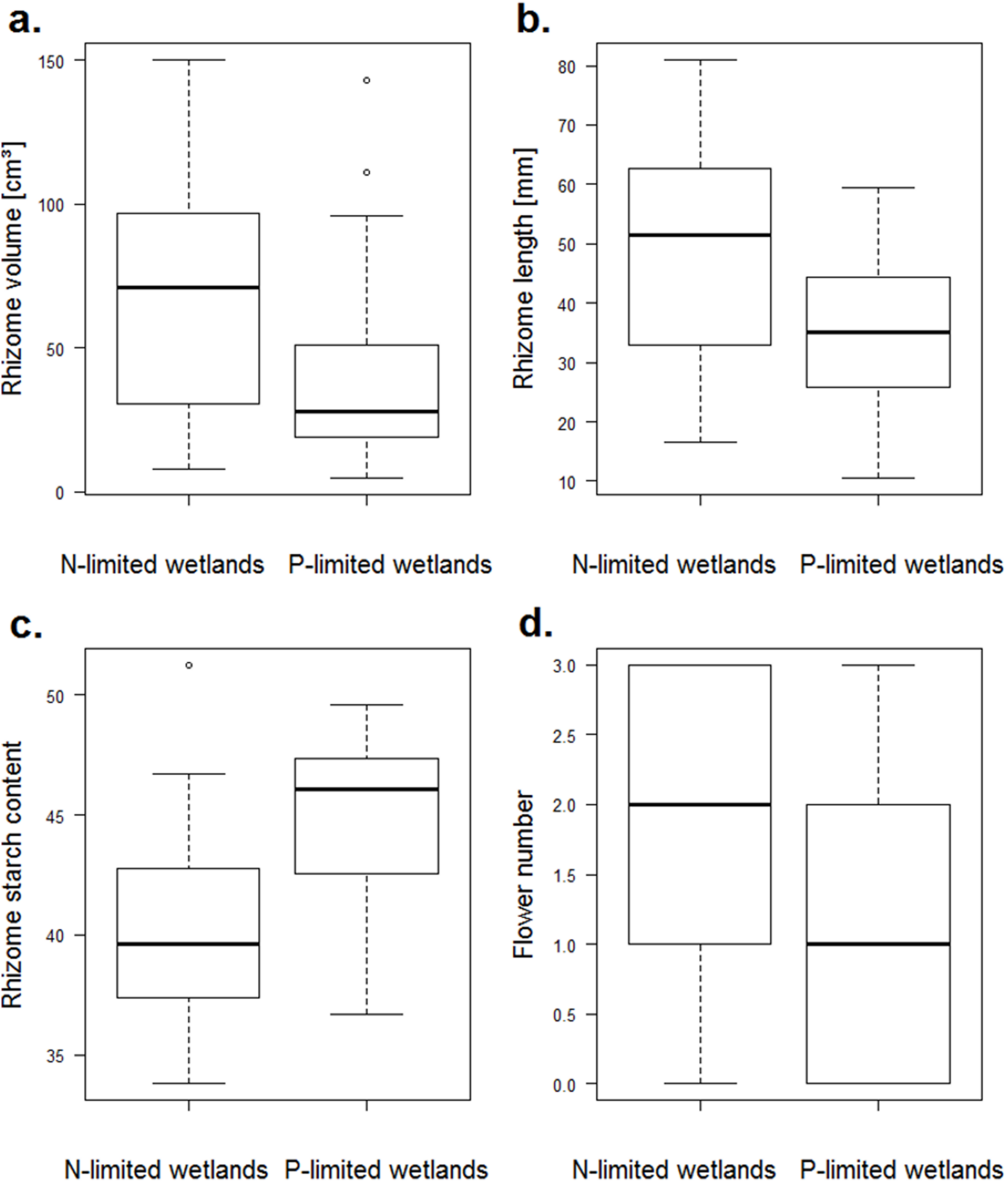
Boxplots of traits of *N. lutea* that differed significantly between the nitrogen- and the phosphorus-limited wetlands according to the *t*-test. (A) Boxplots of the rhizome volume in nitrogen- or phosphorus-limited wetlands (*p*-value = 0.02684). (B) Boxplots of the rhizome length in nitrogen- or phosphorus-limited wetlands (*p*-value = 0.01816). (C) Boxplots of the rhizome starch content in nitrogen- or phosphorus-limited wetlands (*p*-value = 0.01075). (D) Boxplots of the flower number in nitrogen- or phosphorus-limited wetlands (*p*-value = 0.01231).

Among the growth traits, only the rhizome length differed between the nitrogen- and the phosphorus-limited wetlands (*p*-value = 0.018), and it reached higher values for the nitrogen-limited wetlands (Fig. 3). For reproduction traits, the number of flowers was higher for the nitrogen-limited wetlands (*p*-value = 0.012, Fig. 3). Two other reproduction traits (the water content of the fruits and the seed:fruit mass ratio) tended to be higher for nitrogen-limited wetlands, but without reaching significance: the water content of the fruits and the seed:fruit mass ratio (*p*-value = 0.058 and 0.059, respectively). Among the storage traits, both the rhizome volume and starch content differed among the nitrogen- and phosphorus-limited wetlands (*p*-value = 0.027 and 0.011, respectively). The rhizome volume was higher in the nitrogen-limited wetlands, while the starch content was higher in the phosphorus-limited wetlands (Fig. 3). None of the defence traits varied significantly between the nitrogen- and phosphorus-limited wetlands.

After the Holm adjustment of the *p*-values, no relationship remained significant.

## Discussion

### Relationships between wetland physicochemical characteristics and *N. lutea* traits

#### Water resource conditions

The PC1 of the PCA outlined the functional contrast of the resource conditions, and particularly between the phosphorus-rich (positive values along the axis) and the nitrogen-rich (negative values along the axis) aquatic ecosystems. The 11 wetlands were arranged along this resource gradient. The factorial coordinates of wetlands along this axis were strongly correlated to their trophic state index (Carlson, 1977; Table 3).

**Table 3:** Trophic state index (TSI) of Carlson (1977) applied to the 11 studied wetlands, calculated with the chlorophyll-a (Chla) average concentration and the total phosphorus average concentration (TP) of the water. The Trophic State Index of Carlson varies between zero and 100, zero characterising highly oligotrophic water and 100 characterising highly eutrophic water. The significance and location of the wetland codes are indicated in Fig. 1.

Floodplain	Wetland	TSI (Chla)	TSI (TP)	MOY
Ain	SBR	49.1	30.7	39.9
	VILC	44.4	35.3	39.8
Doubs	BING	54.4	57.7	56.0
	CHA	61.3	64.5	62.9
	GRI	53.9	64.7	59.3
	LON	60.3	68.6	64.4
	MER	55.9	37.4	46.6
Loue	BAR	62.1	60.5	61.3
	CHEM	62.3	59.4	60.8
	CDC	41.0	40.2	40.6
	CLO	48.0	42.5	45.3

**Figure 4:**
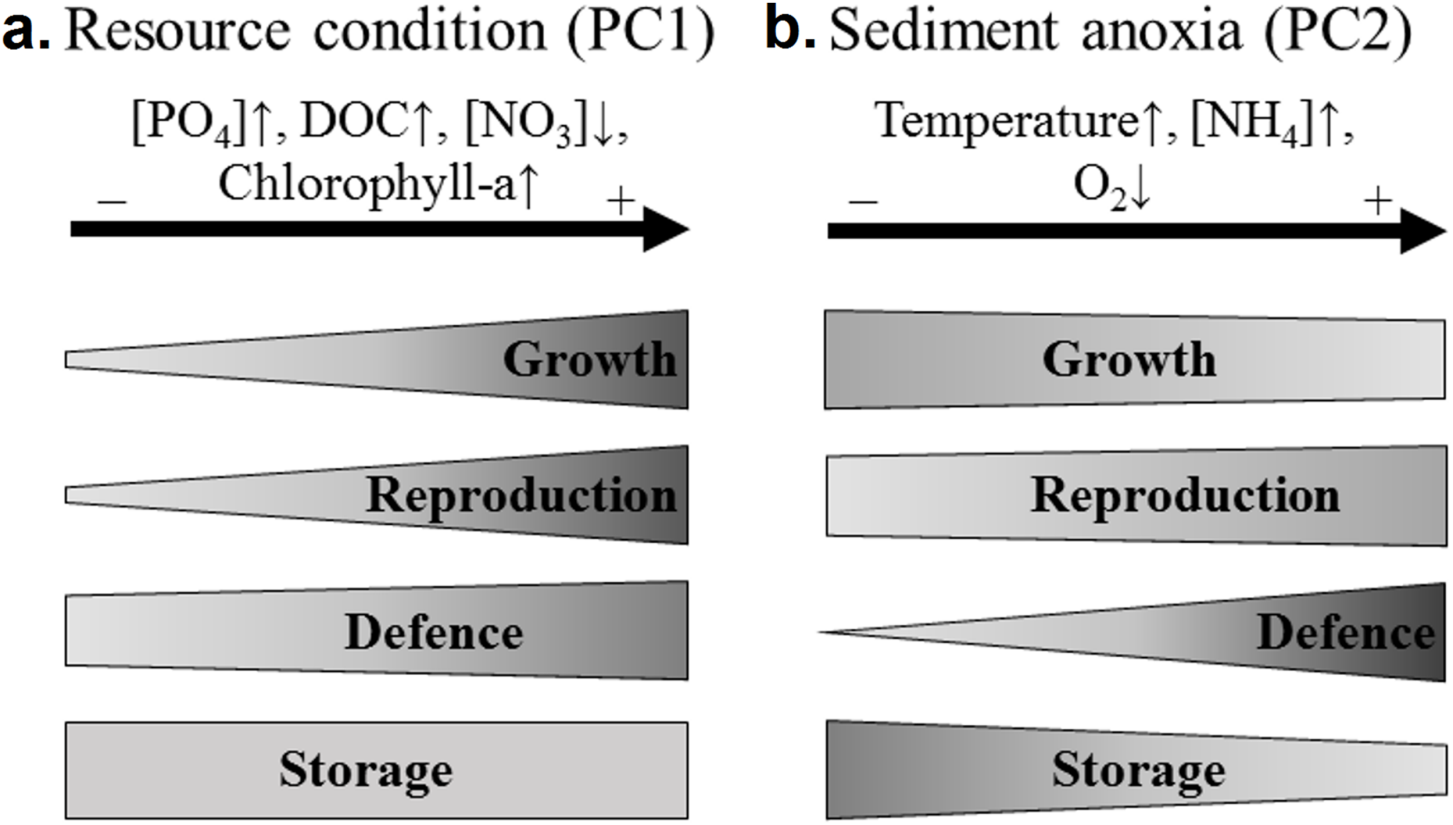
Recapitulative scheme of the variation of *N. lutea* sets of traits in response to environmental variations in the studied wetlands. The shape of the frames reflects the proportion of traits correlated to the environmental factorial axis considered (PC1 or PC2). As discussed below, both rhizome diameter and volume were considered as growth traits in this figure. (A) Three out of four growth traits (i.e. the length, diameter and volume of the rhizome), three out of four reproduction traits (i.e. the seed:fruit mass ratio and both number of seed and flower), one out of three defence traits (i.e. the leaf C:N ratio), and zero out of two storage traits were correlated to PC1 (i.e. the eutrophication axis). (B) One out of four growth traits (i.e. the rhizome length), one out of four reproduction traits (i.e. the seed:fruit mass ratio), three out of three defence traits (i.e. the leaf C:N ratio, the leaf sclereids abundance, and the leaf water content), and one out of two storage traits (i.e. the rhizome water content) were correlated to PC2 (i.e. the sediment anoxia axis).

The four sets of traits responded differently according to the resource conditions (Fig. 4). Concerning growth related traits, the rhizome length was significantly and positively correlated with PC1, that is, the resource conditions. Both rhizome diameter and volume were considered storage traits in our study. Unexpectedly, they increased with the phosphate concentration in water, while storage is usually considered to increase with habitat adversity (Grime, 2006; Grasset et al., 2015). The rhizome volume was significantly higher in phosphorus-rich wetlands and did not correlate with the rhizome starch content (that was higher in phosphorus-poor wetlands). This suggests that diameter and volume may be considered as growth instead of storage traits. The increase in water phosphate concentrations was therefore related to the increase in rhizome growth traits (volume, diameter and length).

Some other growth traits, such as leaf length and leaf area index have previously been demonstrated to increase with water nutrient content and particularly with phosphates (Klok & Van Der Velde, 2017). *N. lutea* is considered to be a top-competitor in standing waters (Arts et al., 1990; Bornette et al., 2008; Bornette & Puijalon, 2011; Grasset et al., 2015). As size traits are frequently related to plant competitive ability (Grime, 2006), decreasing water and sediment nutrient load (especially the phosphate concentration) may decrease *N. lutea* competitive ability.

The number of flowers and seeds were also significantly and positively correlated with the increase in phosphorus and organic carbon in water, demonstrating a positive relationship between these nutrients and the reproduction traits.

Concerning the defence traits, the C:N ratio was significantly and unexpectedly positively correlated with the increase in phosphorus and organic carbon in water. According to the conservation strategy, leaf tissue resistance usually increases when the resource conditions of the habitat decrease (Ordoñez et al., 2009). The observed increase in the C/N ratio may reflect the consequence of an increasing plant size with the increase in phosphorus and organic carbon in water, leading to a higher number or size of leaves and petioles, which requires structurally resistant tissues. Even if starch storage may relate to reserve accumulation for spring sprouting, the present results suggest that it is also related to stress-tolerance (low phosphorus concentrations in water), as structural carbohydrates, which are frequently outlined in the literature (Gebauer, Reynolds & Tenhunen, 1995). This suggests the occurrence of different storage tactics according to plant strategy and/or phenology. Competitive species, such as *N. lutea*, may store starch for both seasonal regeneration and stress tolerance, whereas stress-tolerant species may also store structural carbohydrates for increasing tissue lifespan (Grasset et al., 2015). Finally, *N. lutea*, a late successional species, may be more plastic in its morphological variables than in its metabolic variables.

#### Sediment anoxic stress

The PC2 of the PCA outlined the functional contrast between wetlands with high average water temperatures and high ammonium nitrogen concentrations in water (e.g. MER and CHA) and the other wetlands. The significant and positive correlation between dissolved oxygen and both ammonium and temperature along PC2 may appear counterintuitive. Ammonium is usually oxidised in nitrate in aerobic conditions (Reddy & DeLaune, 2008). In the present study, O_2_ was measured below the water surface and strongly related to the photosynthetic activity. In nutrient-rich ecosystems that have rather high summer temperatures, phytoplankton is usually abundant, leading to O_2_ oversaturation near the water surface during the day. In such a situation, the oxygen concentration frequently decreases with depth and may even become null close to the substrate, even in low-depth ecosystems (Hutchinson, 1975). Therefore, dissolved oxygen is usually negatively correlated with ammonium and temperature at the bottom of such warm and ammonium-rich wetlands (Koko et al., 2017).

The four sets of traits responded differently according to this anoxic stress in the sediment (Fig. 4). Above the four growth-related traits, only the rhizome length was significantly and negatively correlated with the anoxic stress. *N. lutea* has a ventilation system (Dacey, 1981; Dacey & Klug, 1982), but only the tip of the roots leak oxygen (Smits et al., 1990). This ventilation system may be insufficient for preventing the effect of toxic compounds produced in anoxic sediments (e.g. reduced forms of Fe and Mn, ethanol, lactic acid, acetaldehydes and aliphatic acids, Pezeshki, 2001), and may lead to the intrusion of sulphide in rhizomes and meristematic tissues, known to inhibit mitochondrial activity in eukaryotic cells (Pedersen, Binzer & Borum, 2004), leading ultimately to a decrease of plant growth.

The seed:fruit mass ratio was the only reproduction trait significantly related to the ammonium concentration (i.e. negatively). This suggests that an increasing ammonium concentration may increase the seed mass to the detriment of the fruit shell. *N. lutea* may produce seeds with bigger reserves, potentially increasing the ability of seedlings to tolerate high water ammonium concentrations and the related oxygen depletion, increasing their recruitment success (Westoby et al., 1996).

The three defence traits (i.e. the sclereid abundance, and the C:N ratio and water content of the floating leaf blades) were significantly correlated with the PC2, that is, ammonium concentration in water. The sclereid abundance and the C:N ratio of the floating leaf blades were negatively related to PC2, whereas the leaf water content was positively related to PC2. Even if the ammonium ion is the preferred nitrogen source for plant growth (Reddy & DeLaune, 2008), it may become toxic for rooted aquatic plants (Clarke & Baldwin, 2002). Furthermore, high ammonium concentrations, usually accompanied by low oxygen concentrations, favour the production of several phytotoxic compounds in the rhizosphere (Ponnamperuma, 1972; Pezeshki, 2001). In such a stressful context, the decrease in the leaf water content and the increase in the C:N ratio and sclereid abundance may increase both tissue resistance and lifespan (Lamberti-Raverot & Puijalon, 2012; Konno, Inoue & Nakamura, 2014; Klok & Van Der Velde, 2017). Herbivory was not quantified in this study but may also relate to the variation of these three defence traits, especially to the sclereid abundance (Konno, Inoue & Nakamura, 2014). The way this trait is related to grazing pressure may be further explored, as it may be an integrated proxy of grazing pressure on *N. lutea*. Moreover, there may be a strong relationship between temperature, degree of herbivory, turn-over of floating leaf blades and growth of *N. lutea*.

Concerning the storage, our hypothesis that assumed that growth should decrease and storage should increase in cases of habitat adversity (Kubin, Melzer & Čížková, 1994) is partly validated. Indeed, high ammonium concentrations and temperatures in wetlands were significantly and negatively correlated to rhizome growth and biomass production. However, the starch content measured in the rhizome portion produced the year before the rhizome sampling was not significantly related to wetland coordinates along PC2. This suggests either that the storage capacity of *N. lutea* is low in case of increasing ammonium and thermal stress (associated with sediment anoxia), or that the redistribution of reserves between recently produced and older rhizome parts may have influenced the results by underestimating the rhizome starch content.

#### Sediment nutrient loads and N. lutea traits

The sediment characteristics seem less related to *N. lutea* traits than water characteristics. Indeed, only three traits (i.e. number of flowers and seeds, rhizome diameter) were significantly and negatively correlated with the nitrogen concentration throughout the depth of the sediment. Two additional traits (i.e. the rhizome length and volume) were by considering the upper two centimeters of sediment. Sediments with high nitrogen concentrations (and organic carbon concentration), which are usually related to late successional stages in wetlands, may lead to a decrease of trait performance for *N. lutea*. Some indirect effects of organic matter accumulation in aquatic ecosystems, such as the accumulation of toxic and soluble organic carbon compounds in both sediment and interstitial water (Barko & Smart, 1986), may relate to this decrease. This result may partly explain why *N. lutea* decreases in abundance in wetlands at an advanced successional stage toward a march and a terrestrial ecosystem (Barrat-Segretain, 1996; Bornette & Puijalon, 2011). If the rhizome volume and diameter are considered as growth traits as previously suggested, then this accumulation of toxic and soluble organic carbon compounds may act as an inhibitory factor for the growth of *N. lutea*. Among the reproduction traits, the seed:fruit mass ratio was significantly and positively correlated with both nitrogen and organic carbon concentrations in sediment, also suggesting a decrease in plant performance, possibly also related to toxic organic compounds (i.e. less seeds, but bigger reserves, Westoby et al., 1996).

Only five traits (i.e. rhizome length and volume, number of seeds, leaf C:N ratio and seed:fruit mass ratio) were significantly and positively correlated with the phosphorus concentration throughout the depth of the sediment, suggesting that either sediment phosphorus may poorly contribute to plant development, probably because of lower bioavailability compared to water phosphate (Rattray, Howard-Williams & Brown, 1991), or that the species is able to store a large stock of phosphorus in rhizomes (Twilley, Brinson & Davis, 1977; Brock, Van Der Velde & Van de Steeg, 1987), allowing the species to tolerate strong fluctuations in available phosphorus in the habitats.

#### Trait contrast according to the N or P limitation of wetlands

Four traits significantly differed between the nitrogen- and phosphorus-limited ecosystems (i.e. rhizome length volume and starch content, and number of flowers, Fig. 3). Three of these traits (i.e. rhizome length and volume and number of flowers) were higher in phosphorus-rich wetlands (i.e. nitrogen-limited wetlands) and increased with the water phosphorus concentration, outlining the key role of phosphorus on the plant traits of *N. lutea*, contrary to the water nitrogen that seems to poorly affect its phenotypic variations.

Plants increasingly invested in starch accumulation in phosphorus-limited situations (i.e. nitrogen-rich wetlands). Nitrate availability may poorly affect plant storage in comparison to phosphate availability (Kubin, Melzer & Čížková, 1994).

However, these interpretations have to be taken with caution because the significance was not confirmed after the Holm correction of *p*-values.

### Consequences for the ecological niche of *N. lutea*

The variation in *N. lutea* growth and reproduction traits was mostly affected by phosphorus availability, and nitrogen seemed to play a minor role in its phenotypic variations. However, nitrogen was always abundant in the 11 studied wetlands, making it difficult to efficiently measure nitrogen-limitation. Although nitrogen is a secondary nutrient in freshwater ecosystems in comparison to phosphorus (Suttle & Harrison, 1988; Güsewell, Koerselman & Verhoeven, 2003), some studies highlighted alterations in plant traits due to a lack of nitrogen in aquatic ecosystems (Feller et al., 2003).

*Nuphar lutea* is a long-living clonal species that may face huge contrasts in environmental conditions through its life. In such a situation, phenotypic adjustments may be essential for facing environmental variations. Our results highlighted that the performance-related traits of *N. lutea* seemed to be improved by phosphate and/or organic carbon in water, particularly for both growth and reproductive success, at a short time scale (over a year). On the contrary, the performance of *N. lutea* decreases when it faces nutrient stress: its growth and reproductive success seem to be forsaken by the increase of defence traits. The absence of variation in some traits, such as leaf number and fruit water content, which appear unrelated to the measured environmental characteristics, suggests either that the variation in such traits has no consequence for *N. lutea* or that these traits are constrained by *N. lutea* morphology, or that their variation is constrained by environmental variation that occur at larger temporal or spatial scales. For example, *N. lutea* only produces one flower per spiral of leaves each year, and the number of flowers may only increase with the increase in spiral number, that is, with a strong increase in rhizome diameter, which only occurs through over several years of growth. In the same way, the surprising absence of correlation between the rhizome starch content and habitat severity (i.e. high ammonium concentrations and low oxygen concentrations) suggests that the species is unable to store starch in the case of anoxic stress, or that this stress may not be mitigated by reserve accumulation. Either the species is unable to tolerate this stress, or it uses some other strategy (e.g. accumulation of structural carbohydrates in rhizomes, for example, which may explain the decrease of rhizome water content with habitat severity).

*Nuphar lutea* is a top-competitor species (Bornette et al., 2008), generally favoured in nutrient-rich habitats. However, such nutrient-rich habitats may disfavour the species if anoxia becomes so high that the species is unable to counterbalance its negative effect through its oxygenation of the rhizosphere. Global warming and eutrophication should consequently lead to *N. lutea* progression as long as temperature and productivity does not exceed a threshold value beyond which the plant will be unable to maintain a sufficient degree of oxidation of the rhizosphere for its survival.

Adverse conditions may also occur in highly oligotrophic conditions, such as in cold phosphorus-poor wetlands.

Our study aimed as searching the relationships between plant traits and environmental variables in a large set of wetlands over a year study. The fact that *N. lutea* is a long-lived plant species may lead to environment-species traits relationships that occur at a longer time scale than the one year of this study. In the same way, the very large size of rhizomes may lead to an integration of the response to the environmental signal at a very large scale, leading to a possible attenuation of the response measured at the rhizome fragment scale, even if previous studies outlined the reactivity of plant ramets to habitat variability (Hutchings & Price, 1993). This may be true for sediment characteristics, as they may vary strongly both horizontally and vertically.

Even if the environmental characteristics of wetlands were rather contrasting, we may have underestimated the effect of environmental characteristics on plant traits. For example, we did not sample habitats with a low pH, but such habitats may have induced different plant responses (e.g. because of low carbon availability, low production and low mineralisation rate of organic matter, Kok, Van Der Velde & Landsbergen, 1990). In the same way, the temporal scale of the study did not allow to assess the effect of environmental variations that occur over longer time scale. For example, river incision, leading to the oligotrophication and cooling of wetlands along karstic rivers (Bravard et al., 1997), may affect adversely *N. lutea* at a time scale larger than one year, decreasing progressively its health through time.

## Conclusions

The hypotheses of this work were that (1) the environmental variation within our set of wetlands led to significant variation among four sets of traits related respectively to growth, reproduction, defence and storage, and (2) that nutrient limitation should affect plant traits of *N. lutea*. Using innovative methods partly based on rhizome growth traits, this study provides an integrative vision of species success and demonstrates the significant effect of wetland characteristics on *N. lutea* traits. This study outlines the elements of vulnerability of plant populations in more eutrophic and oligotrophic situations. It demonstrates that eutrophication (increase in phosphorus water concentration), per se, is not an element of vulnerability of the species but more the ammonium and associated anoxia linked to a high sediment load in organic matter and high water temperatures. The study suggests that the investment in sclereids may be related to grazing pressure, but the exact role of grazing and dewatering in such production needs to be elucidated. Finally, it outlines the promising way to use the rhizome growth pattern to reconstruct the recent history of *N. lutea* plant populations.

## Supplemental Information

10.7717/peerj.7861/supp-1 Supplemental Information 1 Water physico-chemical characteristics of wetlands, collected each month over a year cycle (12 values per parameter per wetland; mean ± standard deviation). The significance of wetland codes and the wetland location are indicated in Fig. 1.

10.7717/peerj.7861/supp-2 Supplemental Information 2 Sediment chemical characteristics of wetlands (mean of the two and 40 upper centimetres of sediment). The significance of wetland codes and the wetland location are indicated in Fig. 1.

10.7717/peerj.7861/supp-3 Supplemental Information 3 Traits of *Nuphar lutea* sampled in the 11 studied wetlands (mean ± standard deviation). FM, Fresh Mass; DM, Dry Mass. The significance of wetland codes and the wetland location are indicated in Fig. 1.

## Additional Information and Declarations

### Data Availability

The following information was supplied regarding data availability: Wetland functioning and functional traits of *Nuphar lutea* data are available at Gudrun Bornette, Charles Henriot (2019): Wetland functionning and functional traits of *Nuphar lutea*. Chrono-environnement. DOI 10.25666/DATAOSU-2019-02-15. Data is also available at: https://chrono-environnement.univ-fcomte.fr/NUPHAR_WETLAND/.

## References

[ref-1] Abdi H (2010). Encyclopedia of Research Design.

[ref-2] Ackerly DD, Dudley SA, Sultan SE, Schmitt J, Coleman JS, Linder CR, Sandquist DR, Geber MA, Evans AS, Dawson TE (2000). The evolution of plant ecophysiological traits: recent advances and future directions: new research addresses natural selection, genetic constraints, and the adaptive evolution of plant ecophysiological traits. AIBS Bulletin.

[ref-90] Arthaud F, Vallod D, Robin J, Bornette G (2012). Eutrophication and drought disturbance shape functional diversity and life-history traits of aquatic plants in shallow lakes. Aquatic Sciences.

[ref-3] Arthaud F, Vallod D, Robin J, Wezel A, Bornette G (2013). Short-term succession of aquatic plant species richness along ecosystem productivity and dispersal gradients in shallow lakes. Journal of Vegetation Science.

[ref-4] Arts GHP, Van Der Velde G, Roelofs JGM, Van Swaay CAM (1990). Successional changes in the soft-water macrophyte vegetation of (sub)atlantic, sandy, lowland regions during this century. Freshwater Biology.

[ref-5] Barko JW, Adams MS, Clesceri NL (1986). Environmental factors and their consideration in the management of submersed aquatic vegetation: a review. Journal of Aquatic Plant Management.

[ref-6] Barko JW, Smart RM (1986). Sediment-related mechanisms of growth limitation in submersed macrophytes. Ecology.

[ref-7] Barrat-Segretain M-H (1996). Germination and colonisation dynamics of *Nuphar lutea* (L.) Sm. in a former river channel. Aquatic Botany.

[ref-8] Bolpagni R, Pino F (2017). Sediment nutrient drivers of the growth dynamics of the rare fern *Marsilea quadrifolia*. Hydrobiologia.

[ref-10] Bornette G, Puijalon S (2011). Response of aquatic plants to abiotic factors: a review. Aquatic Sciences.

[ref-11] Bornette G, Tabacchi E, Hupp C, Puijalon S, Rostan J-C (2008). A model of plant strategies in fluvial hydrosystems. Freshwater Biology.

[ref-12] Bravard J-P, Amoros C, Pautou G, Bornette G, Bournaud M, Creuzé des Châtelliers M, Gibert J, Peiry J-L, Perrin J-F, Tachet H (1997). River incision in south-east France: morphological phenomena and ecological effects. Regulated Rivers: Research & Management.

[ref-13] Bret-Harte MS, Shaver GR, Zoerner JP, Johnstone JF, Wagner JL, Chavez AS, Gunkelman RF, Lippert SC, Laundre JA (2001). Developmental plasticity allows *Betula nana* to dominate tundra subjected to an altered environment. Ecology.

[ref-14] Brock TCM, Van Der Velde G, Van de Steeg HM (1987). The effects of extreme water level fluctuations on the wetland vegetation of a nymphaeid dominated oxbow lake in The Netherlands. Archiv für Hydrobiologie Beihefte. Ergebnisse der Limnologie.

[ref-15] Carlson RE (1977). A trophic state index for lakes. Limnology and Oceanography.

[ref-16] Chapin FS, Autumn K, Pugnaire F (1993). Evolution of suites of traits in response to environmental stress. American Naturalist.

[ref-17] Clarke E, Baldwin AH (2002). Responses of wetland plants to ammonia and water level. Ecological Engineering.

[ref-18] Coley PD, Bryant JP, Chapin FS (1985). Resource availability and plant antiherbivore defense. Science.

[ref-19] Craine JM, Froehle J, Tilman DG, Wedin DA, Chapin FS (2001). The relationships among root and leaf traits of 76 grassland species and relative abundance along fertility and disturbance gradients. Oikos.

[ref-20] Cronin G, Wissing KD, Lodge DM (1998). Comparative feeding selectivity of herbivorous insects on water lilies: aquatic vs. semi-terrestrial insects and submersed vs. floating leaves. Freshwater Biology.

[ref-21] Dacey JWH (1981). Pressurized ventilation in the yellow waterlily. Ecology.

[ref-22] Dacey JWH, Klug MJ (1982). Ventilation by floating leaves in *Nuphar*. American Journal of Botany.

[ref-23] Dorken ME, Barrett SC (2004). Phenotypic plasticity of vegetative and reproductive traits in monoecious and dioecious populations of *Sagittaria latifolia* (Alismataceae): a clonal aquatic plant. Journal of Ecology.

[ref-24] Du Z-Y, Wang Q-F (2016). Phylogenetic tree of vascular plants reveals the origins of aquatic angiosperms. Journal of Systematics and Evolution.

[ref-25] Feller IC, Whigham DF, McKee KL, Lovelock CE (2003). Nitrogen limitation of growth and nutrient dynamics in a disturbed mangrove forest, Indian River Lagoon, Florida. Oecologia.

[ref-26] Garnier E, Laurent G (1994). Leaf anatomy, specific mass and water content in congeneric annual and perennial grass species. New Phytologist.

[ref-27] Gebauer RLE, Reynolds JF, Tenhunen JD (1995). Growth and allocation of the arctic sedges *Eriohorum angustifolium* and *E. vaginatum*: effects of variable soil oxygen and nutrient availability. Oecologia.

[ref-28] Grasset C, Delolme C, Arthaud F, Bornette G (2015). Carbon allocation in aquatic plants with contrasting strategies: the role of habitat nutrient content. Journal of Vegetation Science.

[ref-29] Grime JP (2006). Plant strategies, vegetation processes, and ecosystem properties.

[ref-30] Große W (1996). Pressurised ventilation in floating-leaved aquatic macrophytes. Aquatic Botany.

[ref-31] Gross KL, Willig MR, Gough L, Inouye R, Cox SB (2000). Patterns of species density and productivity at different spatial scales in herbaceous plant communities. Oikos.

[ref-32] Grosse W, Büchel HB, Tiebel H (1991). Pressurized ventilation in wetland plants. Aquatic Botany.

[ref-33] Güsewell S, Koerselman W, Verhoeven JT (2003). Biomass N:P ratios as indicators of nutrient limitation for plant populations in wetlands. Ecological Applications.

[ref-34] Holm S (1979). A simple sequentially rejective multiple test procedure. Scandinavian Journal of Statistics.

[ref-35] Hutchings MJ, Price EA (1993). Does physiological integration enable clonal herbs to integrate the effects of environmental heterogeneity?. Plant Species Biology.

[ref-36] Hutchinson GE (1975). A treatise on limnology. Volume III. Limnological botany.

[ref-37] Jeppesen E, Peder Jensen J, SØndergaard M, Lauridsen T, Landkildehus F (2000). Trophic structure, species richness and biodiversity in Danish lakes: changes along a phosphorus gradient. Freshwater Biology.

[ref-38] Jump AS, Penuelas J (2005). Running to stand still: adaptation and the response of plants to rapid climate change. Ecology Letters.

[ref-39] Kausch AP, Seago JL, Marsh LC (1981). Changes in starch distribution in the overwintering organs of *Typha latifolia* (Typhaceae). American Journal of Botany.

[ref-40] Keddy P, Gaudet C, Fraser LH (2000). Effects of low and high nutrients on the competitive hierarchy of 26 shoreline plants. Journal of Ecology.

[ref-41] Klok PF, Van Der Velde G (2017). Plant traits and environment: floating leaf blade production and turnover of waterlilies. PeerJ.

[ref-42] Klok PF, Van Der Velde G (2019). Initial decomposition of floating leaf blades of waterlilies: causes, damage types and impacts. PeerJ.

[ref-43] Kok CJ, Van Der Velde G, Landsbergen KM (1990). Production, nutrient dynamics and initial decomposition of floating leaves of *Nymphaea alba* L. and *Nuphar lutea* (L.) Sm. (Nymphaeaceae) in alkaline and acid waters. Biogeochemistry.

[ref-44] Koko S, Irvine K, Jindal R, Thongdara R (2017). Spatial and temporal variations of dissolved oxygen in cha-am municipality wastewater treatment ponds using GIS Kriging interpolation. Journal of Water Management Modeling.

[ref-45] Konno K, Inoue TA, Nakamura M (2014). Synergistic defensive function of raphides and protease through the needle effect. PLOS ONE.

[ref-46] Kornijów R, Measey GJ, Moss B (2016). The structure of the littoral: effects of waterlily density and perch predation on sediment and plant-associated macroinvertebrate communities. Freshwater Biology.

[ref-47] Kouki J (1993). Herbivory modifies the production of different leaf types in the yellow water-lily, *Nuphar lutea* (Nymphaeceae). Functional Ecology.

[ref-48] Kubin P, Melzer A, Čížková H (1994). The relationship between starch content in rhizomes of *Phragmites australis* (Cav.) Trin. ex Steud. and trophic conditions of habitat. Proceedings of the Royal Society of Edinburgh. Section B. Biological Sciences.

[ref-49] Lamberti-Raverot B, Puijalon S (2012). Nutrient enrichment affects the mechanical resistance of aquatic plants. Journal of Experimental Botany.

[ref-50] Lavorel S, Garnier É (2002). Predicting changes in community composition and ecosystem functioning from plant traits: revisiting the Holy Grail. Functional Ecology.

[ref-51] Maberly SC, Madsen TV (2002). Freshwater angiosperm carbon concentrating mechanisms: processes and patterns. Functional Plant Biology.

[ref-52] Maberly SC, Spence DHN (1989). Photosynthesis and photorespiration in freshwater organisms: amphibious plants. Aquatic Botany.

[ref-53] McDonald AJS, Lumsden PJ, Nicholas JR, Davies WJ (1994). Nutrient supply and plant growth. Physiology, Growth and Development of Plants in Culture.

[ref-54] Nakamura M, Nakamura T, Tsuchiya T, Noguchi K (2013). Functional linkage between N acquisition strategies and aeration capacities of hydrophytes for efficient oxygen consumption in roots. Physiologia Plantarum.

[ref-55] Ordoñez JC, Van Bodegom PM, Witte J-PM, Wright IJ, Reich PB, Aerts R (2009). A global study of relationships between leaf traits, climate and soil measures of nutrient fertility. Global Ecology and Biogeography.

[ref-56] Padgett DJ (2007). A monograph of *Nuphar* (Nymphaeaceae). Rhodora.

[ref-57] Padgett DJ, Les DH, Crow GE (1999). Phylogenetic relationships in *Nuphar* (Nymphaeaceae): evidence from morphology, chloroplast DNA, and nuclear ribosomal DNA. American Journal of Botany.

[ref-92] Pedersen O, Binzer T, Borum J (2004). Sulphide intrusion in eelgrass (*Zostera marina* L.). Plant, Cell and Environment.

[ref-58] Pezeshki SR (2001). Wetland plant responses to soil flooding. Environmental and Experimental Botany.

[ref-59] Ponnamperuma FN (1972). The chemistry of submerged soils. Advances in Agronomy.

[ref-60] Poorter H, De Jong ROB (1999). A comparison of specific leaf area, chemical composition and leaf construction costs of field plants from 15 habitats differing in productivity. New Phytologist.

[ref-61] Puijalon S, Bornette G (2004). Morphological variation of two taxonomically distant plant species along a natural flow velocity gradient. New Phytologist.

[ref-62] Puijalon S, Bornette G, Sagnes P (2005). Adaptations to increasing hydraulic stress: morphology, hydrodynamics and fitness of two higher aquatic plant species. Journal of Experimental Botany.

[ref-63] Puijalon S, Bouma TJ, Douady CJ, Van Groenendael J, Anten NP, Martel E, Bornette G (2011). Plant resistance to mechanical stress: evidence of an avoidance-tolerance trade-off. New Phytologist.

[ref-64] Puijalon S, Piola F, Bornette G (2008). Abiotic stresses increase plant regeneration ability. Evolutionary Ecology.

[ref-91] R Core Team (2017). R: a language and environment for statistical computing.

[ref-65] Rascio N (2002). The underwater life of secondarily aquatic plants: some problems and solutions. Critical Reviews in Plant Sciences.

[ref-66] Rattray MR, Howard-Williams C, Brown JMA (1991). Sediment and water as sources of nitrogen and phosphorus for submerged rooted aquatic macrophytes. Aquatic Botany.

[ref-67] Reddy KR, Agami M, Tucker JC (1990). Influence of phosphorus on growth and nutrient storage by water hyacinth (*Eichhornia crassipes* (Mart.) Solms) plants. Aquatic Botany.

[ref-68] Reddy KR, DeLaune RD (2008). Biogeochemistry of wetlands: science and applications.

[ref-69] Ribaudo C, Bartoli M, Longhi D, Castaldi S, Neubauer SC, Viaroli P (2012). CO2 and CH4 fluxes across a *Nuphar lutea* (L.) Sm. stand. Journal of Limnology.

[ref-70] Rodier J, Bazin C, Broutin J, Chambon P, Champsaur H, Rodi L (1996). L’Analyse de l’Eau.

[ref-71] Sahli HF, Conner JK (2011). Testing for conflicting and nonadditive selection: floral adaptation to multiple pollinators through male and female fitness. Evolution.

[ref-72] Santamaría L (2002). Why are most aquatic plants widely distributed? Dispersal, clonal growth and small-scale heterogeneity in a stressful environment. Acta Oecologica.

[ref-73] Santamaría L, Van Vierssen W (1997). Photosynthetic temperature responses of fresh- and brackish-water macrophytes: a review. Aquatic Botany.

[ref-74] Schoelynck J, Bal K, Verschoren V, Penning E, Struyf E, Bouma T, Meire D, Meire P, Temmerman S (2014). Different morphology of *Nuphar lutea* in two contrasting aquatic environments and its effect on ecosystem engineering. Earth Surface Processes and Landforms.

[ref-75] Smits AJM, De Lyon MJH, Van Der Velde G, Steentjes PLM, Roelofs JGM (1988). Distribution of three nymphaeid macrophytes (*Nymphaea alba* L., *Nuphar lutea* (L.) Sm. and *Nymphoides peltata* (Gmel.) O. Kuntze) in relation to alkalinity and uptake of inorganic carbon. Aquatic Botany.

[ref-76] Smits AJM, Laan P, Thier RH, Van Der Velde G (1990). Root aerenchyma, oxygen leakage patterns and alcoholic fermentation ability of the roots of some nymphaeid and isoetid macrophytes in relation to the sediment type of their habitat. Aquatic Botany.

[ref-77] Smolders AJP, Lamers LPM, Lucassen E, Van Der Velde G, Roelofs JGM (2006). Internal eutrophication: how it works and what to do about it—a review. Chemistry and Ecology.

[ref-78] Stenberg JA, Stenberg JE (2012). Herbivory limits the yellow water lily in an overgrown lake and in flowing water. Hydrobiologia.

[ref-79] Strand VV, Weisner SE (2002). Interactive effects of pressurized ventilation, water depth and substrate conditions on *Phragmites australis*. Oecologia.

[ref-80] Suttle CA, Harrison PJ (1988). Ammonium and phosphate uptake rates, N:P supply ratios, and evidence for N and P limitation in some oligotrophic lakes. Limnology and Oceanography.

[ref-81] Titus JE, Sullivan PG (2001). Heterophylly in the yellow waterlily, *Nuphar variegata* (Nymphaeaceae): effects of [CO_2_], natural sediment type, and water depth. American Journal of Botany.

[ref-82] Tolivia D, Tolivia J (1987). Fasga: a new polychromatic method for simultaneous and differential staining of plant tissues. Journal of Microscopy.

[ref-83] Twilley RR, Brinson MM, Davis GJ (1977). Phosphorus absorption, translocation, and secretion in *Nuphar luteum*^1^. Limnology and Oceanography.

[ref-84] Urbas P, Zobel K (2000). Adaptive and inevitable morphological plasticity of three herbaceous species in a multi-species community: field experiment with manipulated nutrients and light. Acta Oecologica.

[ref-85] Van Der Velde G, Custers CPC, De Lyon MJH (1986). The distribution of four nymphaeid species in the Netherlands in relation to selected abiotic factors.

[ref-86] Weiner J (2004). Allocation, plasticity and allometry in plants. Perspectives in Plant Ecology, Evolution and Systematics.

[ref-87] Wells CL, Pigliucci M (2000). Adaptive phenotypic plasticity: the case of heterophylly in aquatic plants. Perspectives in Plant Ecology, Evolution and Systematics.

[ref-88] Westoby M, Leishman M, Lord J, Poorter H, Schoen DJ (1996). Comparative ecology of seed size and dispersal. Philosophical Transactions of the Royal Society of London. Series B: Biological Sciences.

[ref-89] Yentsch CS, Menzel DW, Elsevier BV (1963). A method for the determination of phytoplankton chlorophyll and phaeophytin by fluorescence. Deep Sea Research and Oceanographic Abstracts.

