# Supplementary material for: Relationships between key functional traits of the waterlily Nuphar lutea and wetland nutrient content
Source: PeerJ. 2019 Oct 17;7:e7861. doi: 10.7717/peerj.7861 (PMC6800984; doi:10.7717/peerj.7861)
Supplement: Supplemental Information 1 — The significance of wetland codes and the wetland location are indicated in Fig. 1. [file peerj-07-7861-s001.doc]

**Table S1** Water physico-chemical characteristics of wetlands, collected each month over a year cycle (12 values per parameter per wetland; mean ± standard deviation). The significance of wetland codes and the wetland location are indicated in figure 1.

| **Wetland** | **Average Depth (m)** | **Dewatering**  **(dry in summer)** | **Water temperature (°C)** | **pH** | **Saturated O_2_**  **(%)** | **[P-PO_4_^3-^] (mg.l^-1^)** | **[N-NH_4_] (mg.l^-1^)** | **[N-NO_3_] (mg.l^-1^)** | **Chlorophyll-a (mg.l^-1^)** | **Organic carbon (mg.l^-1^)** | **Conductiviy (µS/cm)** |
| --- | --- | --- | --- | --- | --- | --- | --- | --- | --- | --- | --- |
| **SBR** | 0.666 | No | 14.2  ±5.3 | 7.43  ±0.17 | 58.2  ±24.2 | 0.006  ±0.012 | 0.021  ±0.009 | 4.99  ±1.24 | 2.2  ±2.2 | 3.62  ±2.74 | 588.9  ±30.9 |
| **VILC** | 0.729 | Yes | 11.9  ±1.9 | 7.39  ±0.20 | 57.4  ±18.55 | 0.013  ±0.020 | 0.008  ±0.007 | 2.27  ±0.58 | 4.4  ±6.2 | 4.37  ±3.55 | 502.8  ±26.0 |
| **BING** | 1.461 | No | 12.8  ±5.0 | 7.46  ±0.22 | 58.0  ±19.9 | 0.040  ±0.048 | 0.038  ±0.059 | 2.00  ±1.64 | 11.4  ±13.0 | 4.59  ±1.94 | 473.5  ±113.8 |
| **CHA** | 0.472 | No | 14.5  ±6.2 | 7.70  ±0.34 | 95.1  ±46.8 | 0.064  ±0.072 | 0.039  ±0.039 | 1.17  ±1.53 | 23.3  ±38.6 | 3.41  ±1.21 | 526.3  ±96.8 |
| **GRI** | 0.5375 | Yes | 13.4  ±6.3 | 7.46  ±0.28 | 74.7  ±35.9 | 0.065  ±0.091 | 0.036  ±0.047 | 1.30  ±1.31 | 10.8  ±11.1 | 8.48  ±11.59 | 554.5  ±141.6 |
| **LON** | 0.5615 | Yes | 14.5  ±6.3 | 7.54  ±0.40 | 87.6  ±42.5 | 0.087  ±0.108 | 0.030  ±0.050 | 0.80  ±1.19 | 20.6  ±23.3 | 6.91  ±3.53 | 512.6  ±67.5 |
| **MER** | 0.977 | No | 15.1  ±7.1 | 7.63  ±0.22 | 81.4  ±24.5 | 0.008  ±0.008 | 0.038  ±0.034 | 0.69  ±0.66 | 13.3  ±12.4 | 4.72  ±3.71 | 562.9  ±41.4 |
| **BAR** | 0.603 | No | 13.7  ±2.8 | 7.51  ±0.18 | 84.6  ±26.1 | 0.048  ±0.039 | 0.020  ±0.036 | 2.44  ±0.51 | 24.8  ±44.8 | 2.37  ±0.62 | 478.0  ±21.3 |
| **CDC** | 0.4425 | Yes | 13.1  ±3.8 | 7.31  ±0.21 | 79.7  ±13.7 | 0.011  ±0.017 | 0.015  ±0.022 | 2.94  ±0.53 | 3.0  ±3.9 | 1.89  ±0.95 | 533.3  ±30.8 |
| **CHEM** | 0.248 | Yes | 13.5  ±5.7 | 7.61  ±0.49 | 82.6  ±20.1 | 0.046  ±0.043 | 0.020  7±0.029 | 0.84  ±1.11 | 25.6  ±36.2 | 5.62  ±2.34 | 453.5  ±38.4 |
| **CLO** | 0.165 | No | 16.1  ±5.5 | 7.60  ±0.43 | 96.8  ±49.3 | 0.014  ±0.017 | 0.027  ±0.044 | 1.11  ±0.89 | 5.9  ±7.7 | 6.07  ±8.43 | 492.3  ±70.8 |
