# Supplementary material for: Relationships between key functional traits of the waterlily Nuphar lutea and wetland nutrient content
Source: PeerJ. 2019 Oct 17;7:e7861. doi: 10.7717/peerj.7861 (PMC6800984; doi:10.7717/peerj.7861)
Supplement: Supplemental Information 2 — The significance of wetland codes and the wetland location are indicated in Fig. 1. [file peerj-07-7861-s002.doc]

**Table S2** Sediment chemical characteristics of wetlands (mean of the 2 and 40 upper centimetres of sediment). The significance of wetland codes and the wetland location are indicated in figure 1.

|  | **2 upper cm of sediment** | | | **40 upper cm of sediment** | | |
| --- | --- | --- | --- | --- | --- | --- |
| **Wetland** | **Total phosphorus (%)** | **Total nitrogen (%)** | **Total organic carbon (%)** | **Total phosphorus (%)** | **Total nitrogen (%)** | **Total organic carbon (%)** |
| **SBR** | 0.054 | 0.004 | 0.043 | 0.040 | 0.004 | 0.043 |
| **VILC** | 0.083 | 0.007 | 0.082 | 0.055 | 0.006 | 0.079 |
| **BING** | 0.124 | 0.008 | 0.066 | 0.087 | 0.005 | 0.045 |
| **CHA** | 0.294 | 0.005 | 0.043 | 0.271 | 0.005 | 0.040 |
| **GRI** | 0.163 | 0.007 | 0.065 | 0.092 | 0.005 | 0.044 |
| **LON** | 0.134 | 0.007 | 0.074 | 0.082 | 0.004 | 0.037 |
| **MER** | 0.153 | 0.011 | 0.090 | 0.096 | 0.009 | 0.070 |
| **BAR** | 0.108 | 0.006 | 0.059 | 0.096 | 0.006 | 0.057 |
| **CHEM** | 0.140 | 0.006 | 0.059 | 0.132 | 0.005 | 0.049 |
| **CDC** | 0.174 | 0.013 | 0.104 | 0.092 | 0.011 | 0.093 |
| **CLO** | 0.129 | 0.012 | 0.149 | 0.075 | 0.008 | 0.096 |
