# Supplementary material for: Relationships between key functional traits of the waterlily Nuphar lutea and wetland nutrient content
Source: PeerJ. 2019 Oct 17;7:e7861. doi: 10.7717/peerj.7861 (PMC6800984; doi:10.7717/peerj.7861)
Supplement: Supplemental Information 3 — FM, Fresh Mass; DM, Dry Mass. The significance of wetland codes and the wetland location are indicated in Fig. 1. [file peerj-07-7861-s003.doc]

**Table S3** Traits of *Nuphar lutea* sampled in the 11 studied wetlands (mean ± standard deviation). FM: Fresh Mass, DM: Dry Mass. The significance of wetland codes and the wetland location are indicated in figure 1.

| **Wetland** | **Rhizome volume (cm^3^)** | **Rhizome water content (% FM)** | **Rhizome diameter (mm)** | **Rhizome length (mm)** | **Rhizome starch content (% DM)** | **C:N ratio of plant tissue** | **Sclereids (number/ cm²)** | **Leaf water content**  **(% FM)** | **Leaf number produced per rhizome apex per year** | | **Number of flowers produced per rhizome apex per year** | **Number of seeds per fruit** | **Fruit water content (% FM)** | **Seed:fruit mass ratio** |
| --- | --- | --- | --- | --- | --- | --- | --- | --- | --- | --- | --- | --- | --- | --- |
| **SBR** | 25.25  ±3.95 | 87.38  ±4.98 | 33.26  ±4.73 | 29.63  ±7.19 | 42.25  ±2.65 | 15.01  ±0.98 | 92.14  ±20.20 | 87.68  ±1.41 | 9.50  ±2.38 | 1.25  ±1.50 | | 89.80  ±106.43 | 0.87  ±0.02 | 0.25  ±0.17 |
| **VILC** | 40.16  ±20.34 | 85.57  ±1.47 | 32.74  ±8.09 | 46.20  ±6.76 | 34.47  ±5.93 | 11.04  ±0.71 | 35.82  ±13.57 | 90.33  ±0.54 | 10.20  ±0.45 | 0.60  ±0.89 | | 40.67  ±21.55 | 0.89  ±0.01 | 0.02  ±0.01 |
| **BING** | 58.27  ±56.46 | 85.15  ±1.82 | 42.11  ±10.53 | 38.38  ±13.35 | 46.30  ±3.06 | 15.59  ±0.61 | 111.39  ±31.43 | 87.85  ±1.54 | 10.80  ±2.95 | 1.00  ±1.00 | | 288.00  ±133.13 | 0.89  ±0.01 | 0.18  ±0.10 |
| **CHA** | 73.81  ±36.02 | 84.85  ±3.22 | 39.48  ±7.44 | 55.88  ±13.82 | 44.73  ±7.29 | 16.37  ±1.61 | 98.65  ±67.76 | 87.78  ±1.61 | 11.25  ±1.89 | 1.75  ±1.26 | | 304.70  ±126.32 | 0.87  ±0.02 | 0.43  ±0.13 |
| **GRI** | 28.77  ±22.55 | 84.94  ±1.14 | 33.90  ±7.91 | 27.40  ±10.56 | 38.53  ±1.14 | 14.59  ±1.77 | 124.74  ±13.96 | 87.33  ±0.93 | 8.60  ±0.89 | 1.20  ±1.10 | | 178.10  ±98.54 | 0.86  ±0.02 | 0.36  ±0.15 |
| **LON** | 80.15  ±22.32 | 85.23  ±0.56 | 42.64  ±3.48 | 55.40  ±8.44 | 42.01  ±1.42 | 15.39  ±1.43 | 100.98  ±29.01 | 88.13  ±0.87 | 10.80  ±1.92 | 2.40  ±0.55 | | 267.22  ±94.73 | 0.84  ±0.01 | 0.52  ±0.13 |
| **MER** | 32.89  ±26.59 | 87.73  ±1.98 | 36.03  ±7.50 | 28.30  ±10.10 | 44.11  ±1.90 | 18.70  ±2.50 | 156.94  ±35.15 | 86.38  ±2.40 | 8.40  ±1.52 | 1.40  ±1.34 | | 177.30  ±125.46 | 0.87  ±0.02 | 0.49  ±0.14 |
| **LON** | 80.15  ±22.32 | 85.23  ±0.56 | 42.64  ±3.48 | 55.40  ±8.44 | 42.01  ±1.42 | 15.39  ±1.43 | 100.98  ±29.01 | 88.13  ±0.87 | 10.80  ±1.92 | 2.40  ±0.55 | | 267.22  ±94.73 | 0.84  ±0.01 | 0.52  ±0.13 |
| **BAR** | 56.55  ±24.92 | 83.92  ±1.28 | 42.38  ±8.80 | 38.50  ±3.66 | 47.89  ±1.14 | 13.26  ±1.43 | 79.94  ±46.38 | 88.28  ±2.09 | 9.40  ±2.41 | 1.80  ±0.45 | | 299.90  ±107.97 | 0.87  ±0.01 | 0.22  ±0.12 |
| **CHEM** | 146.64  ±4.91 | 86.03  ±2.33 | 48.80  ±8.76 | 68.75  ±17.32 | 35.18  ±2.20 | 17.30  ±1.52 | 98.34  ±53.83 | 88.36  ±0.71 | 11.50  ±2.12 | 2.50  ±0.71 | | 326.60  ±138.07 | 0.88  ±0.02 | 0.22  ±0.13 |
| **CDC** | 43.93  ±38.13 | 84.60  ±2.74 | 31.39  ±9.97 | 48.90  ±7.92 | 40.57  ±4.79 | 15.51  ±0.87 | 109.55  ±61.09 | 88.24  ±1.54 | 11.20  ±1.30 | 0.40  ±0.89 | | 153.50  ±64.07 | 0.87  ±0.01 | 0.36  ±0.12 |
| **CLO** | 13.56  ±8.98 | 85.11  ±0.45 | 27.03  ±5.06 | 21.86  ±7.99 | 44.96  ±3.03 | 13.06  ±0.75 | 90.03  ±26.73 | 88.18  ±0.36 | 9.20  ±2.05 | 0.40  ±0.89 | | 170.70  ±67.62 | 0.88  ±0.01 | 0.30  ±0.06 |
